# Aestivation of the African Malaria Mosquito, *Anopheles gambiae* in the Sahel

**DOI:** 10.4269/ajtmh.2010.09-0779

**Published:** 2010-09

**Authors:** Tovi Lehmann, Adama Dao, Alpha Seydou Yaro, Abdoulaye Adamou, Yaya Kassogue, Moussa Diallo, Traoré Sékou, Cecilia Coscaron-Arias

**Affiliations:** Laboratory of Malaria and Vector Research, National Institute of Allergy and Infectious Diseases, National Institutes of Health, Rockville, Maryland; Malaria Research and Training Center, University of Bamako, Bamako, Mali

## Abstract

The African malaria mosquito, *Anopheles gambiae*, inhabits diverse environments including dry savannas, where surface waters required for larval development are absent for 4–8 months per year. Under such conditions, *An. gambiae* virtually disappears. Whether populations survive the long dry season by aestivation (a dormant state promoting extended longevity during the summer) or are reestablished by migrants from distant locations where larval sites persist has remained an enigma for over 60 years. Resolving this question is important, because fragile dry season populations may be more susceptible to control. Here, we show unequivocally that *An. gambiae* aestivates based on a demographic study and a mark release–recapture experiment spanning the period from the end of one wet season to the beginning of the next. During the dry season, *An. gambiae* was barely detectable in Sahelian villages of Mali. Five days after the first rain, before a new generation of adults could be produced, mosquito abundance surged 10-fold, implying that most mosquitoes were concealed locally until the rain. Four days after the first rain, a marked female *An. gambiae* s.s. was recaptured. Initially captured, marked, and released at the end of the previous wet season, she has survived the 7-month-long dry season. These results provide evidence that *An. gambiae* persists throughout the dry season by aestivation and open new questions for mosquito and parasite research. Improved malaria control by targeting aestivating mosquitoes using existing or novel strategies may be possible.

## Introduction

Malaria causes over 1 million deaths every year, most of which are African children. One important reason that this burden is so heavy in Africa is that the malaria-transmission machinery consists of an exceptionally robust vectorial system including *Anopheles gambiae* s.s. (representing two incipient species known as the M and S molecular forms), a sibling species *An. arabiensis*, and *An. funestus*. These vectors exploit diverse environments including expansive dry savannas and semi-desert areas, where the surface waters required for larval development disappear for 4–8 months each year. Extreme seasonal fluctuation is a hallmark of the population dynamics of these malaria vectors, especially in arid environments where these anophelines apparently disappear during the dry season but population sizes rapidly increase after the onset of rains.^1^–^3^ Eggs, larvae, and pupae of *An. gambiae* cannot withstand desiccation over a few days,^4^–^7^ and adults seldom survive beyond 2 months.^8^,^9^ Therefore, a mosquito that survives over 3 months during the dry season when no larval sites are available can be defined as aestivating.^10^,^11^ Aestivation is a recurring state of summer dormancy, typically characterized by suppressed reproduction and/or growth that facilitate extended survival during harsh conditions. Temperate mosquito species commonly undergo winter diapause,^12^–^16^ but summer diapause is virtually unknown in mosquitoes. Many insects, including those inhabiting tropical regions, undergo seasonal dormancy,^17^,^18^ but whether *An. gambiae* aestivates is one of the longest-lasting mysteries of malariology. Several studies reported finding a few mosquitoes during the late dry season when no larval sites could be found,^10^,^19^–^21^ but resolving if these mosquitoes survived throughout the long dry season (aestivation) or whether they migrated from areas with ongoing permanent breeding has not been possible. To date, only two studies provided evidence for aestivation. Omer and Cloudsley-Thompson^22^ found low density of *An. arabiensis* adults 20 km away from the Nile in Sudan when no larval sites were available. During the early dry season, adult females (mostly collected in houses and wells) did not develop eggs despite feeding on blood. This physiological condition, known as gonotrophic dissociation, is consistent with aestivation. In the laboratory, *An. arabiensis* survived during the dry season for 206 days,^11^ whereas *An. gambiae* s.l. from Bobo Dioulasso, Burkina Faso survived in the laboratory up to 150 days.^10^ However, similar studies could not replicate these results^5^,^19^,^23^–^25^ and thus, cast doubt about the generality of these findings. Furthermore, estimates of survival in nature have never matched these records. Population genetics studies aimed at detecting dry-season bottlenecks in vector populations have found evidence for the contrary. Surprisingly large effective population size (N_e_ ~ 2,000) of *An. arabiensis* was estimated in the Sahel and dry savannas,^21^,^26^ as was the case (N_e_ ~ 6,500) for *An. gambiae* s.s. in wet savannas,^27^ suggesting a large aestivating population or mass migration.^28^

To test the hypothesis that *An. gambiae* aestivates in the Sahel, we undertook a mosquito demographic study including a mark release–recapture (MRR) study conducted from one rainy season (September to November 2008) to the beginning of the next (April to June 2009) in the village Thierola, located in the Sahelian belt of Mali. In the course of this experiment, a total of 6,931 *An. gambiae* s.l. were captured, marked, and released, each with a unique identification code based on a combination of up to four dots of nine colors placed on four positions on the body.

## Materials and Methods

The study was performed in Thierola (13.40° N, 7.13° W) (Table 1), a small village (276 inhabitants living in a total of 120 houses) 3 km away from its nearest neighboring village, Zanga (Table 1), and 6 km from the next closest neighboring village, Bako (Table 1). The rectangular mud-brick, mud-roof houses of the Bambara ethnic group (80% of the population) are clustered together in adjacent compounds. The circular, mud-brick, thatch-roof houses of the Fulani ethnic group (20% of the population) are organized in five compounds separated by 200 m from each other, along an arc 500 m south of the main village. The community grows primarily millet, sorghum, maize (corn), and peanuts during the rainy season (June to September). Cattle, sheep, goats, and chickens are raised by most families. The rains fill two large ponds and numerous small puddles, but usually, all surface waters dry by November. From November until May, rainfall is altogether absent or negligible (total precipitation < 30 mm). After the harvest (October to November), the fields surrounding the village lay bare. Water is only available in four deep wells (~30 m deep). Annual precipitation is approximately 500 mm (513 mm in Segou, which lies 30 km south and 100 km east of Thierola and 380 mm in Nara, which lies 170 km north of Thierola). The natural vegetation consists of grasses, low shrubs, and scattered trees. Only few refugia, such as holes in baobab trees, can be found in a radius of 2 km around the village.

The first phase of the study was a multiple capture, mark, release, and recapture experiment performed during the end of the wet season (mid-September to mid-November 2008). The second and third phases consisted of identical surveys conducted at the end of the subsequent dry season before the first rains and immediately after the first rains, respectively; however, mosquitoes captured in these phases were not released. Live collection by aspiration from all houses (*N* = 120, including abandoned houses and those used for kitchens, storage, and animals), outdoor clay-pot traps, and emergence traps (set over natural larval sites) was conducted every other day during phase I. Live collection in every house was carried out by two trained collectors, each searching for mosquitoes for 10–15 minutes (and until no mosquitoes were being collected for 3–5 minutes). Mosquitoes were released ~12 hours after capture within 5 m from their point of capture. A total of 2,397 male and 4,534 female *An. gambiae* s.l. were captured, marked, and released—each with a unique identification code based on a combination of two to four dots of nine possible colors placed on four positions on the body (three on the ventral side of the abdomen and one on the mesothorax) following methods previously described.^29^ Each mosquito was anesthetized in diethyl ether, examined under dissecting scope, and unless found to be marked, was marked before being placed in a cup and provided with 5% sugar water until release (9:00–11:00 pm). During phase I, a total of 248 mosquitoes (58 males and 190 females) were recaptured, some of which were recaptured two or three times.

During phases II and III, extensive surveys were conducted in Thierola, including daily live adult collections by aspiration from all houses (*N* = 120) as described above. Collections were also performed outdoors in clay pots (*N* = 28) and Centers for Disease Control and Prevention (CDC) traps (*N* = 20) baited with an open fruit (mango/melon) set for 6 nights. Fruit-baited large traps (*N* = 8, set for 6 nights), well traps (*N* = 8, set for 10 nights), and toilet traps (*N* = 8, set for 6 nights) were checked every 3 hours from 6:00 pm to 6:00 am. These traps consisted of standard bed nets (without insecticide) hung above the well or toilet pit, leaving no space for mosquitoes to enter or exit, whereas an approximately 30-cm gap above ground was left for mosquitoes to enter to the fruit-baited traps. Human landing catch was performed by 16 collection sites (8 indoors and 8 outdoors) from 6:00 pm to 6:00 am for 6 nights. Additionally, large (50 cm diameter) and small (15 cm diameter) artificial oviposition sites (*N* = 8 and *N* = 20, respectively) were maintained and surveyed for over 3 weeks in Thierola and Zanga. Finally, extensive surveys of possible larval sites in an area of 20 km diameter around Thierola were conducted over 5 days. Pyrethrum spray collection was conducted after 12 days of live collection in Thierola and Zanga. Similar pyrethrum spray catches were performed in neighboring villages.

## Results

**Figure 1.**
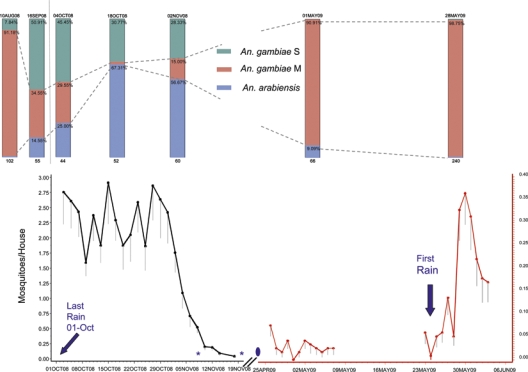
Abundance (**Bottom**) and composition (**Top**) of *An. gambiae* from the late wet season (2008) to the early subsequent wet season (2009). Density is measured by daily live indoor density per house (**Bottom**). The left axis (black) corresponds to the data from the wet season, and the right axis (red) corresponds to the data from the dry season (note difference in scale). Vertical gray lines depict one standard error of the mean density per house. Composition of *An. gambiae* is shown in bars based on pooled collections representing 1- to 18-day intervals centered on the date shown above the bars. Sample size used in calculation of composition is shown beneath each bar, and percentage of each population is given inside the bars. Stars indicate day of desiccation of the last larval site in the village (November 8) and 1.5 km away (November 25), which was the last larval site in a radius of 6 km or more from Thierola. Blue ellipse indicates day of scattered rains (March 22) during the dry season.

Extreme seasonal variation in abundance and composition of *An. gambiae* s.l. is one of its hallmarks, especially in drier environments.^2^,^5^,^8^,^23^,^30^,^31^ Rapid population growth over a few generations, however, is distinct from a rapid surge of density within one generation. Therefore, we measured population density on a daily basis during the end of the wet season (October to November 2008), the end of the dry season (April to May 2009), and the early part of the subsequent wet season (May to June 2009), providing a comprehensive description of the population dynamics. In early November, mosquitoes almost vanished from Thierola (Figure 1). Although scattered rains fell on March 22, 2009 larval sites only had water for up to 6 days, which is insufficient time for development of an egg into an adult (9–11 days); moreover, no larvae were found in those larval sites. Adult density dropped 100-fold from its wet season levels. However, a few *An. gambiae* and *An. arabiensis* could be collected until the end of the dry season (May) (Figure 1 and Table 1). At that time, females comprised the majority of *An. gambiae* s.l. specimens (44 females versus 8 males). All females were collected shortly after taking a blood meal (32% fed, 29% semigravid, and 39% gravid), and most were inseminated (86%). All mosquitoes were collected indoors, except a single female that was collected from human landing catch and one male that was collected in a CDC light trap baited with fruit. Despite having no available larval sites, egg developed normally with little evidence for gonotrophic dissociation (mature or maturing ovaries were observed in all but 5 of 28 gravid females dissected).

By the end of the dry season, the S form apparently disappeared, and the M form predominated (90%) (Figure 1), whereas the rest consisted of *An. arabiensis* (Figure 1). The mosquitoes found during the dry season in neighboring villages in a radius of 20 km from Thierola had similarly low densities and composition (Table 1). Extensive surveys revealed a single small larval site in a dry streambed in the village Filanibougou, 16 km from Thierola, which dried out 2 days later. Adult mosquito density in that village was comparable with that of Thierola (Table 1), suggesting that it too could not serve as a source of migrants.

**Figure 2.**
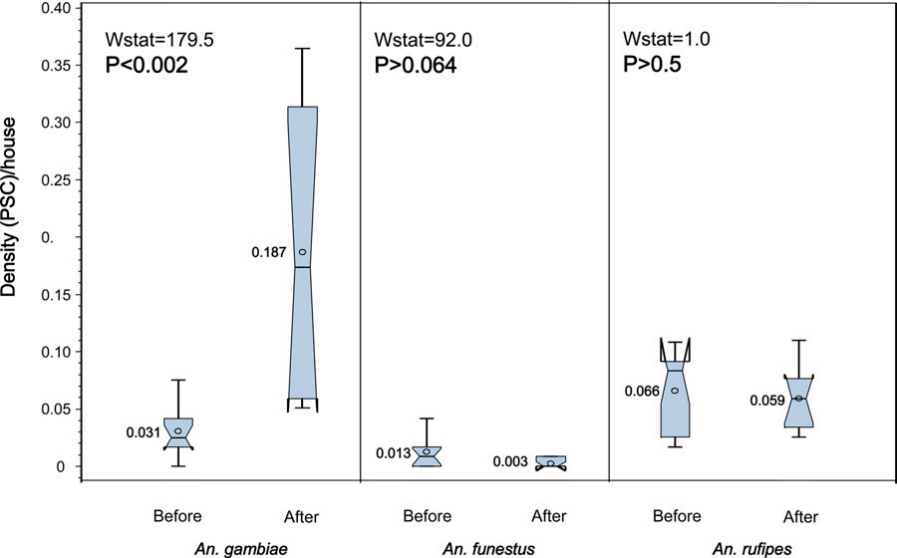
House density of *An. gambiae*, *An. Funestus*, and *An. rufipes* before and after the first rain (May 24, 2009) measured by the total daily live collections indoors in all 120 houses of Thierola. In a box-whisker plot, the box extends between the 25th and 75th percentiles [i.e., across one interquartile range (IQR)], and the whiskers extend up to the most extreme value but not beyond 1.5 times the IQR. Values located over 2.5 IQR from the median are shown. Non-overlapping notched belts indicate significant difference between means (*P* < 0.05). This figure appears in color at www.ajtmh.org.

The first rain fell on May 24, 2009 and filled many larval sites, some of which remained with water for over 3 weeks without additional rain. Five days after the rain, *An. gambiae* density shot up and peaked on the seventh day at a level (0.36/house) 10-fold higher than that during the weeks preceding the rain (mean/house = 0.031) (Figures 1 and 2). Because embryonic and larval development takes at least 9 days, these adults must have emerged before the rain. The surge in density 5–7 days after the rain consisted almost exclusively of M form of *An. gambiae* (237 of a total of 240 collected in Thierola and Zanga after the rain) (Figure 1). Importantly, a surge was not observed in *An. funestus* or *An. rufipes* that coexisted during the dry season (Figure 2), indicating that the surge could not be attributed to collection efficiency.

**Figure 3.**
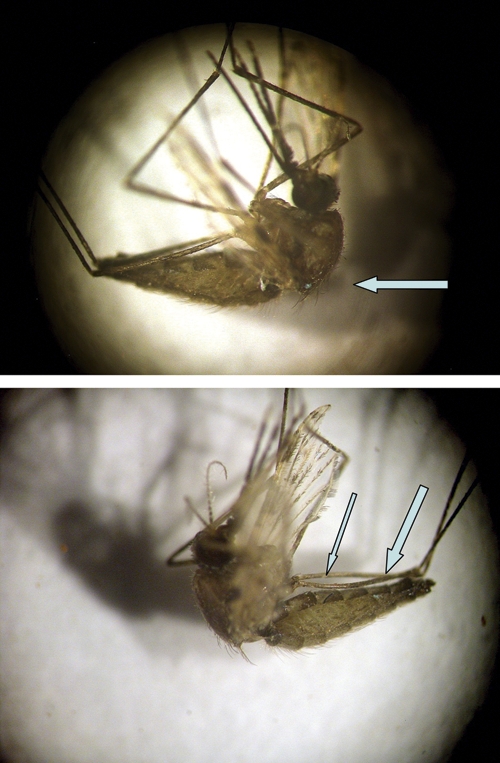
Photographs of the aestivating female recaptured after the first rains nearly 7 months (212 days) after being marked. The painted dot on the dorsal side of the thorax (**Top**) and the two dots on the ventral side of the abdomen (**Bottom**) are visible. Thick arrows point to light-blue paint dots that are clearly visible on the image. The narrow arrow points to the white paint dot that is faintly visible on the image (contrast and brightness of the images were optimized using GIMP2 by Dr. Nick Manoukis).

Every mosquito collected from April to June was inspected for markings painted during the previous wet season (September to November). On the fourth day after the rain (May 27, 2009), a marked female mosquito was recaptured indoors (Figure 3). Originally captured, marked, and released on October 29, 2008 (570 m away from her recapture point), she had survived from the end of the wet season until the beginning of the next wet season, which provides definite evidence for aestivation as a mechanism that allows *An. gambiae* to persist during the dry season in the Sahel.

## Discussion

The long-lasting enigma of whether *An. gambiae* aestivates in arid environments or migrates from neighboring locations where breeding continues has remained unresolved for over 60 years, because the key findings in support of aestivation, namely extended longevity (over 3 months)^10^,^11^ and gonotrophic dissociation,^11^ have not been reproduced despite repeated attempts.^5^,^19^,^23^–^25^ Moreover, extended longevity under laboratory settings^10^,^11^ may not be relevant to natural conditions. The current results, however, resolve this problem by providing definitive evidence for aestivation, confirming the early studies^10^,^11^ and conjectures made on the basis of ecological^28^,^32^–^35^ and genetic data.^21^,^26^,^27^

The recapture of a marked (M form) female at the beginning of the wet season after her release 7 months earlier at the end of the previous wet season is unequivocal evidence for aestivation. Placed in the context of mosquito MRR experiments, it is rare to recapture more than two to three mosquitoes 1 week after the release of thousands.^34^,^36^,^37^ Therefore, a realistic expectation for the number to be recaptured after 7 months cannot be much larger than one. The surge of M-form adults 5–7 days after the first rain, before a new generation of adults could be produced, also attests to the fact that these adults were hidden in the vicinity of the village. A surge was not observed in *An. funestus* or *An. rufipes* (or other member of the *An. gambiae* complex) that coexisted during the dry season (Figure 2), attesting to the fact that the surge could not be attributed to higher collection efficiency. Consistently, the mosquitoes found during the dry season were mostly M form. They probably were residents rather than migrants, because neighboring villages could not serve as a source of migrants given the low densities of *An. gambiae* throughout the area (Table 1) and the isolation of these Sahelian villages. Notably, dispersal over 2–3 km is very rare, even during the wet season.^31^,^34^,^36^,^37^ The concordance in molecular form between the population that persisted throughout the dry season, the surge of adults 5 days after the rain, and the identity of the marked 7-month-old female together provides strong evidence that the M form of *An. gambiae* undergoes aestivation.

Despite finding *An. arabiensis* at the end of the dry season, it remains unclear if it too aestivates. The S form apparently vanished from Thierola during the dry season (between November and May) (Figure 1), and no resurgence of the S form was detected up to 10 days after the first rain. Nonetheless, one S form female appeared in Thierola 9 days after the rain (Figure 1), suggesting that aestivation in this form is rare rather than absent (yet, being unmarked, migration from a distant source cannot be ruled out). Likewise, it is unknown how *An. funestus* (and the zoophilic *An. rufipes*) persists throughout the long dry season, because all known semi-permanent water with submerged vegetation disappears by December. Possibly, the capacity for aestivation is widespread in these anophelines, despite being so elusive for induction in laboratory conditions.

Accordingly, in a typical Sahelian village, hundreds of mosquitoes may aestivate throughout the dry season, hidden in as of yet unknown sites. Aestivating females take blood meals at a lower frequency than non-aestivating females, which is the case for other mosquitoes where diapause is known to occur,^12^,^13^,^38^,^39^ accounting for the low density indoors over the dry season. These findings prompt new investigations on such topics as aging in adult insects, summer diapause in mosquitoes, the molecular basis, physiology, ecology, and population variation in aestivation, and the effect of aestivation on *Plasmodium* and malaria transmission as well as on novel approaches for malaria control by targeting aestivating mosquitoes.

Aestivation may not be the only strategy malaria mosquitoes use to survive the dry season in arid environments. The specific contribution of aestivation to the persistence of populations inhabiting dry areas throughout the dry season should be evaluated in future studies. That larvae were collected from Filanibougou (M form of *An. gambiae* and *An. arabiensis*) in April (above) indicates that some females break their aestivation more readily than others. Gonotrophic dissociation was not observed in our study. This seems to be contradictory to aestivation, but it was also observed at the end of the dry season in Sudan in the same population that exhibited gonotrophic dissociation earlier in the dry season^11^,^22^ as well as by *An. gambiae* s.l. maintained in the laboratory for over 4 months in Burkina Faso.^10^ Notably, no anopheline in Thierola and Zanga laid eggs in the artificial larval sites constructed there (nor in the larval sites formed by the scattered rain in March), although larvae of *Culex quinquefasciatus* inhabited most of these sites. Furthermore, after the first rain, larvae were scarce for 10 days in larval sites that held water for over 3 weeks, suggesting that females reappeared from their refugia but awaited additional signals before breaking aestivation.

Finding that aestivation is key to the persistence of vector populations in arid environments may hold promise for millions of people. For example, long-lasting insecticides applied indoors during the dry season could greatly diminish the numbers of aestivating mosquitoes that would survive to seed the next wet-season generation, thus delaying the build-up of populations after the rains and cutting malaria transmission. During the dry season, mosquitoes were found in all compounds of Thierola, without apparent clustering (data not shown), suggesting that their hiding places are numerous. Insecticide applications during the dry season, therefore, should include all houses. Because aestivating mosquitoes are probably found in every village where larval sites are unavailable for over 3 months, interruption of aestivation must cover large clusters of villages to prevent migration from untreated villages nearby. It is unclear if such a strategy will also reduce malaria transmission where the dry season is shorter.

## Figures and Tables

**Table 1:** House density and composition of *An. gambiae* s.l. in villages around Thierola before (upper rows) and after (lower rows) the first rain

Village	Distance (km) to Thierola/Niger (geoposition coordinates)	Date (2009)	*An. gambiae* s.l. density (houses sprayed)	*An. gambiae* s.l. composition M/S/A (%)‡
Before rain				
Thierola*	0/45 (13.40°N, 7.13°W)	May 8	0.11**^y^** (120)	100/0/0
Zanga*†	3/48 (13.41°N, 7.13°W)	May 4	0.04**^y^** (102)	100/0/0
Bako†	6/49 (13.39°N, 7.16°W)	May 6	0.06**^y^** (50)	100/0/0
Filanibougou	16/52 (13.47°N, 7.08°W)	May 5	0.16**^y^** (50)	75/0/25
After rain				
Thierola*	0/45 (13.40°N, 7.13°W)	June 4	0.31**^y^** (120)	95/0/5
Zanga*†	3/48 (13.41°N, 7.13°W)	June 3	0.12**^y^** (111)	100/0/0
Bako†	6/49 (13.39°N, 7.16°W)	June 3	0.42**^y^** (48)	100/0/0
Kondo	14/59 (13.47°N, 7.15°W)	June 9	0.09**^y^** (67)	75/0/25
Serimana	16/31 (13.32°N, 7.09°W)	June 9	0.23**^y^** (65)	100/0/0
Kolimana	46/0 (13.21°N, 6.56°W)	June 8	1.29**^z^** (55)	100/0/0

Significantly different density values are marked by different letters (*P* < 0.05; Dunnett *T* test comparing all values with that of Thierola after analysis of variance). Note that the spray collection in Thierola was performed after the surge in density, when numbers were in decline (Figure 1).

* Spray catch sampling (presented in this table) is known to provide higher estimates than aspiration of individual live mosquitoes (presented in Figures 1–3).

† Zanga and Bako are the two nearest villages to Thierola.

‡ The relative percentage of the M and S molecular forms of *An. gambiae* (M/S) and *An. arabiensis* (A) are ordered accordingly.
